# TMS Over the Cerebellum Interferes with Short-term Memory of Visual Sequences

**DOI:** 10.1038/s41598-018-25151-y

**Published:** 2018-04-30

**Authors:** C. Ferrari, Z. Cattaneo, V. Oldrati, L. Casiraghi, F. Castelli, E. D’Angelo, T. Vecchi

**Affiliations:** 1 Department of Psychology, University of Milano-Bicocca, Milan, 20126 Italy; 2 IRCCS Mondino Foundation, Pavia, Pavia, 27100 Italy; 3 Department of Brain and Behavioral Sciences, University of Pavia, Pavia, 27100 Italy

## Abstract

Growing evidence suggests that the cerebellum is not only involved in motor functions, but it significantly contributes to sensory and cognitive processing as well. In particular, it has been hypothesized that the cerebellum identifies recurrent serial events and recognizes their violations. Here we used transcranial magnetic stimulation (TMS) to shed light on the role of the cerebellum in short-term memory of visual sequences. In two experiments, we found that TMS over the right cerebellar hemisphere impaired participants’ ability to recognize the correct order of appearance of geometrical stimuli varying in shape and/or size. In turn, cerebellar TMS did not affect recognition of highly familiar short sequences of letters or numbers. Overall, our data suggest that the cerebellum is involved in memorizing the order in which (concatenated) stimuli appear, this process being important for sequence learning.

## Introduction

Recent literature indicates that the cerebellum plays a significant role in the perceptual and cognitive domain, in addition to its established role in motor planning and behavior^1–4^. For instance, converging findings suggest that the cerebellum contributes to the recognition of (recurrent) temporal and spatial relations among stimuli. Indeed, cerebellar lesions impair the ability to recognize serial events as sequences and to identify correct vs. violated sequences of different kind (e.g., verbal, spatial and action sequences)^5–13^. Schubotz and von Cramon’s study^14^ indicates that the cerebellum is also a critical structure in identifying serial events and their violations. Participants in this study attended to the size-based sequential order of a series of circles and had to detect possible sequence violations, whereas in the control task they had just to indicate whether the same circles had same or different color. Results indicate that the cerebellum responded preferentially when the order of the stimuli was attended (sequence task) rather than when the color was. Accordingly, Bubic and colleagues^15^ observed preferential activation of the cerebellum while processing serial stimuli, with the posterior cerebellum being especially activated during detection of sequence violations.

The hypothesized role of the cerebellum in perceptual sequential patterns’ processing originates from the motor domain where the cerebellum has been repeatedly proven to underpin memory of movements’ sequences^1^. Indeed, cerebellar patients did not show implicit (motor) learning for repeated patterns while performing tasks requiring to repeat on a keyboard the sequences of visual stimuli appearing on the screen^16–18^. Accordingly, neuroimaging evidence shows consistent activation in the cerebellum during several tasks involving sequences: visual-motor learning^19^, learning of sequences of hand keypresses^20^ and learning alternating wrist flexion and extension movements^21^. Crucially, the cerebellum seems to be differentially engaged in the successive phases of motor sequence learning, with predominant cerebellar contribution during sequence acquisition rather than sequence execution^1^. Indeed, once a motor sequence is learned (hence it is automatically performed), the cerebellum shows lower activity in comparison to when the sequence has not been learnt as yet^1,21^. In this regard, it has been suggested that the cerebellum sustains gradual learning of motor sequences by implementing and testing internal feedforward models^22^. Accordingly, the effects of cerebellar lesions upon motor skills may also depend on suboptimal implementation of these models rather than exclusively on failures of motor control *per se*^4^.

Brain stimulation evidence also supports a role of the cerebellum in the acquisition of motor sequences^18^. However, no brain stimulation studies have directly investigated whether cerebellar structures are also involved in processing perceptual sequences. Here we addressed this issue, by specifically investigating using transcranial magnetic stimulation (TMS) whether the cerebellum is involved in maintaining in short-term memory the order in which stimuli appear. Indeed, sequence learning relies on the ability to maintain in memory the temporal characteristics of events^23,24^. In two experiments, we asked participants to memorize new sequences of geometrical shapes (Experiment 1 and 2) or to detect violations in highly familiar alphabetic or numerical strings (Experiment 1) while TMS was delivered over the cerebellum, the visual cortex and the vertex (control condition). The familiarity of the sequences was manipulated in order to directly test whether the cerebellum plays a different role in relation to the learning phase of the to-be-processed input^21^. Indeed, there is evidence that the cerebellum is more activated when the structure of the motor pattern has not been completely understood^16,17,19,25^ compared to when the motor patterns are consolidated in memory. Therefore, we expected cerebellar TMS to affect memory of unfamiliar sequences (where acquisition of the sequence is still occurring), and to have no effect on the monitoring of familiar (well known) sequences. TMS was delivered over the right cerebellar hemisphere (rather than medial portions, i.e., vermis) in light of consistent evidence suggesting that perceptual and cognitive processing occurs more in lateral vs. medial cerebellar structures^4,26^.

## Experiment 1

### Method

#### Participants

Eighteen Italian students (3 males, mean age = 21.7 years, *SD* = 1.5) participated in the experiment. Each participant filled in a questionnaire^27^ to evaluate their compatibility with TMS before undergoing the experiment. None of the participants reported neurological problems or history of seizures. None was taking medications that could interfere with neuronal excitability. Written informed consent was obtained from all participants before the experiment. The protocol was approved by the psychological ethical committee of the University of Pavia and participants were treated in accordance with the Declaration of Helsinki.

#### Stimuli and Procedure

Participants were seated comfortably at a distance of 57 cm from a 17″ (1024 × 768 pixels resolution) TFT-LCD computer monitor and wore earplugs to minimize TMS click sound interference. They were required to detect possible irregularities of unfamiliar visual sequences composed by geometrical shapes (*new sequence* condition) and alphabetic or numerical visual sequences (*familiar sequence* condition).

**Figure 1:**
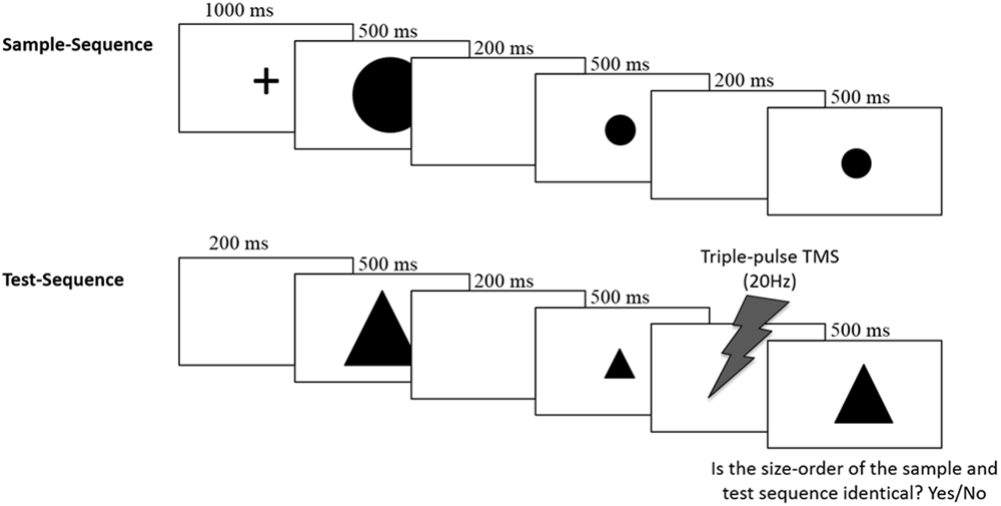
The timeline of an experimental trial in *new sequence* condition of Experiment 1. Participants were required to indicate whether stimuli of the *test* and *sample* sequences followed the same size-order. In the example shown here this was not the case, with the *test* sequence violating the predictable order. TMS was delivered 150 ms before the onset of the last item of the *test* sequence.

In the *new sequence* condition, stimuli consisted of 3 visual shapes (circles, triangles, squares), each appearing in three different sizes throughout the experiment: small (approx. 2*2 deg of visual angle), medium (approx. 4*4 deg) and large (approx. 8*8 deg). The timeline of an experiment trial is presented in Fig. 1. Each trial consisted of a *sample* sequence and of a *test* sequence. Circles were only used in *sample* sequences, triangles and squares were only used in *test* sequences (we used different shapes in the *test* compared to the *sample* sequences to avoid any possible visual adaptation effect). The *sample* sequence consisted of the consecutive presentation of three circles varying in size (e.g., small-large-medium; small-small-large). Each *sample* sequence started with a central fixation cross (presented for 1000 ms), followed by the first item of the series (500 ms duration), a blank screen (200 ms), the second item (500 ms), a blank screen (200 ms), and the third item (500 ms). The *sample* sequence was immediately followed by the *test* sequence. In the *test* sequence either 3 triangles or 3 squares were presented. Stimuli duration was the same as in the *sample* sequence except for the latest stimulus of the sequence that was visible until response. The first two shapes of the *test* sequence were always identical in size to those of the *sample* sequence, whereas the size of the last shape could be the same as that used in the *sample* sequence (i.e., *sample* and *test* were identical in terms of size-sequence) or different (violation of the *sample* sequence). Three shapes of the same size were never presented in the same sequence; furthermore, sequences organized in entirely ascending or descending order (i.e., small-medium-large; large-medium-small) were not used. Participants were instructed to indicate as quickly as possible whether the *test* size-sequence was identical to the *sample* one by left/right key pressing using their right hand (with response keys counterbalanced among participants). This condition consisted of 72 trials (half in which the *test* size-sequence was identical to the *sample* one, and half in which it was different).

*Familiar* sequences consisted of either 4 letters or 4 digits (in the 1–9 range), presented in black ink, 16-point Calibri font and measured approx. 4*4 deg of visual angle. The first three items of the sequence were always presented in correct consecutive order, that is ascending for digits (e.g., 3-4-5) and alphabetic for letters (e.g., L-M-N). The fourth item could be either the correct consecutive item of the sequence (e.g., 3 4 5 6) or the incorrect (e.g., 3 4 5 1): when incorrect, half of them represented the item preceding in the series (as in the example above) and the other half represented the item following in the series. In the *familiar sequence* condition, only *test* sequences were presented (with time parameters identical to those of the *new sequence* condition) and there were no *sample* sequences (since sequences were already known). This condition consisted of 40 trials presented in random order, half in which the correct sequence order was respected, and half in which it was violated. A fixation cross (1000 ms) was presented during the intertrial interval.

For both types of sequence condition, TMS was delivered before the onset of the last stimulus (the last stimulus of the *test* sequence of the *new* sequences where possible violations occurred and the last stimulus of the *familiar* sequences). The experiment consisted of six experimental blocks, one for each TMS site (TMS over the right cerebellum, TMS over the early visual cortex and TMS over the vertex, see below) and sequence condition (*new* and *familiar sequence* condition). A short practice session was presented at the beginning of the experiment to familiarize participants with the stimuli used. Order of TMS site and sequence condition was counterbalanced across participants.

#### Transcranial Magnetic Stimulation (TMS)

Online neuronavigated TMS was performed with a Magstim Rapid^2^ stimulator (Magstim Co., Ltd, Whitland, UK) connected to a 70-mm butterfly coil. At the beginning of each session single pulse TMS was applied at increasing intensities to determine individual motor threshold (MT). MT was defined as the lowest TMS intensity capable of evoking a muscle twitch in the controlateral hand in 5/10 consecutive trials^28,29^. During the experiment, participants were stimulated at 100% of their MT (mean TMS intensity delivered: 56.1% of the maximum stimulator output, SD = 2.9%). No participants reported phosphene perception.

**Figure 2:**
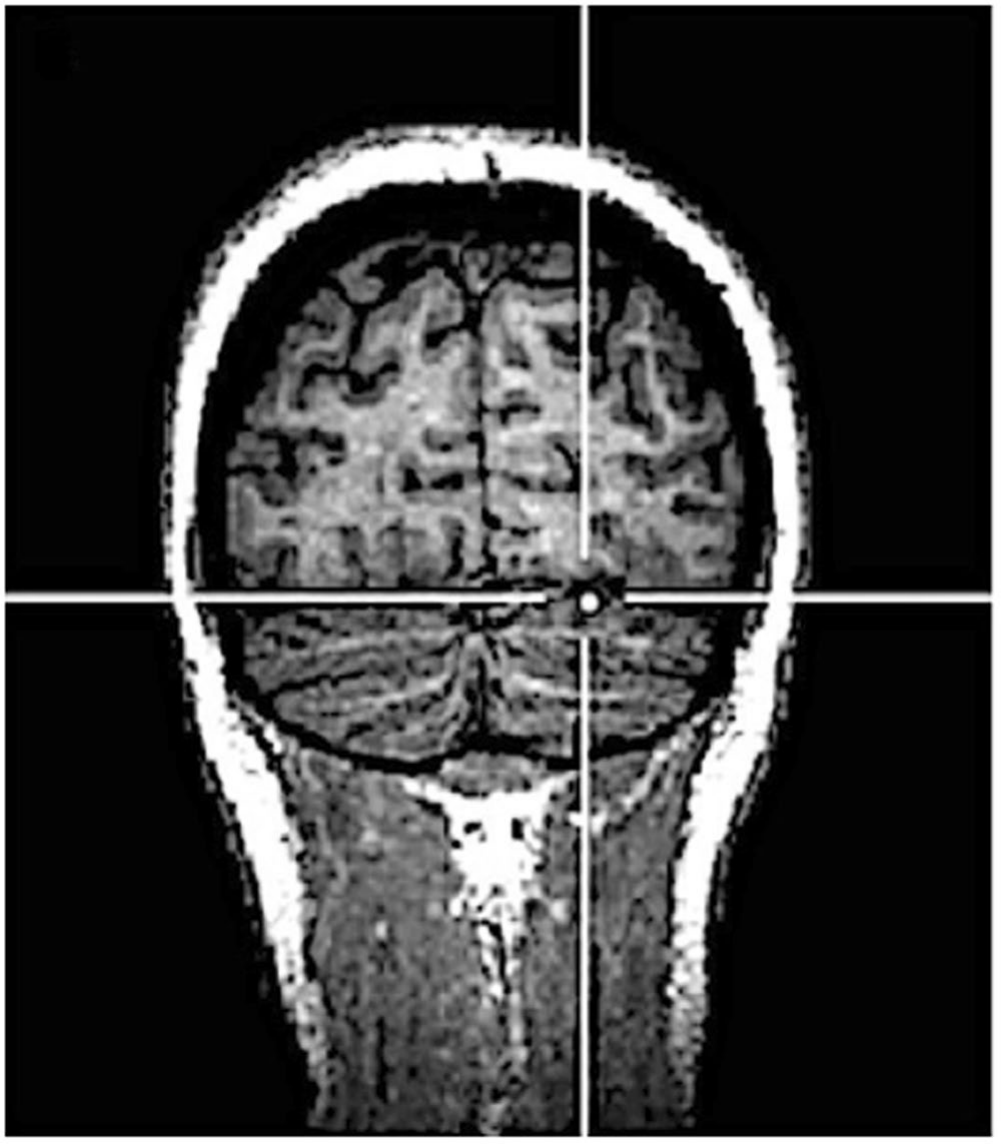
Anatomical Talairach coordinates of the targeted cerebellar site (x = 22, y = −75, z = −21).

Triple-pulse 20 Hz TMS was delivered 150 ms before the presentation of the last stimulus of the *test* sequence (so that the last TMS pulse was given 50 ms before the onset of the stimulus), with similar parameters of stimulation used in previous TMS studies targeting the cerebellum^28,30–32^. TMS was delivered over the right cerebellum, the early visual cortex and the vertex (control condition). Early visual cortex was chosen as additional control area since prior evidence suggests that cerebellar stimulation may spread to primary visual cortex^33^. The cerebellum and the early visual cortex were localized by means of stereotaxic navigation on individual estimated magnetic resonance images (MRI) obtained through a 3D warping procedure fitting a high-resolution MRI template with the participant’s scalp model and craniometric points (Softaxic, EMS, Bologna, Italy). This procedure has been proven to ensure a global localization accuracy of about 5 mm, a level of precision closer to that obtained using individual MRI scans^34^, and has been successfully used in many prior studies^35–37^. Anatomical Talairach coordinates^38^ used for neuronavigation were x = 22, y = −75, z = −21 for the right cerebellum (corresponding to cerebellar loci of activation reported in a previous neuroimaging study investigating processing of visual sequences violation^15^, see Fig. 2) and x = −2, y = −81.4, z = 1.4 for early visual cortex^39^. The region effectively affected by cerebellar TMS corresponded to the superficial layers of the cerebellar cortex^40,41^: in fact, deeper sites were unlikely to be directly reached by the stimulation as the intensity of the electrical field induced by TMS drops dramatically as a function of the distance from the coil^42^. The vertex was localized as the point falling half the distance between the nasion and the inion on the same^43,44^ midline. The coil was placed tangentially to the scalp and held parallel to the midsagittal line, with the handle pointing backward in the vertex and with the handle pointing superiorly in the cerebellum and early visual cortex TMS stimulation.

### Data Availability

All data collected in this experiment are available upon request.

### Results

**Figure 3:**
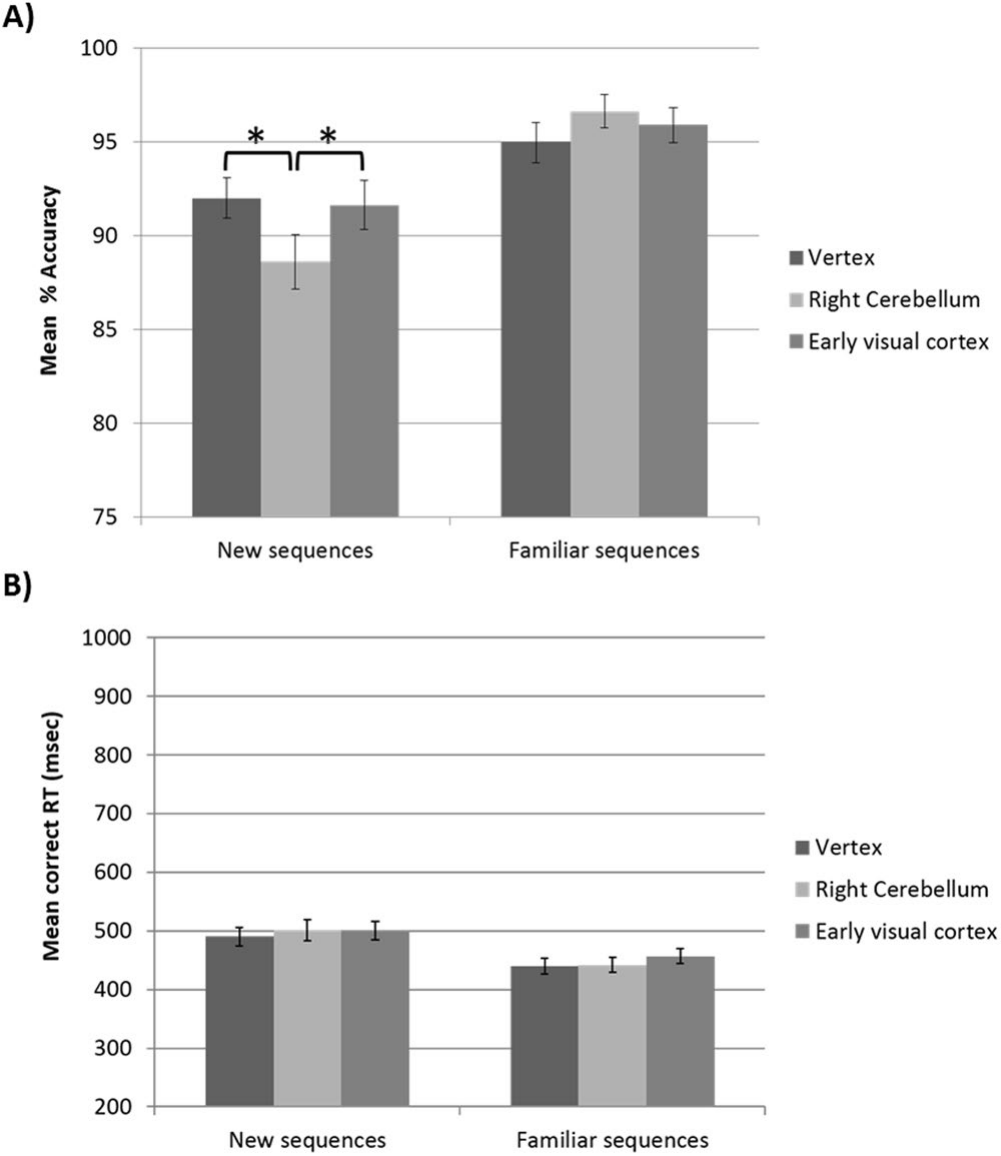
(**A**) Mean percentage accuracy scores and (**B**) mean correct RT as a function of TMS site (right cerebellum, early visual cortex and vertex) and task condition (*new* vs. *familiar sequences*) in Experiment 1. TMS over the cerebellum selectively impaired participants’ accuracy compared to early visual cortex and vertex stimulation in memory for *new* but not *familiar* sequences. RT were not affected by TMS. Error bars represent ±SEM. Asterisks indicate significant differences between conditions (*p < 0.05).

Mean accuracy rates and mean reaction times (RT) for correct responses were computed for each participant in each TMS condition, and are shown in Fig. 3. Data were submitted to repeated-measures ANOVAs with task condition (*new vs*. *familiar sequences*) and TMS (cerebellum, early visual cortex and vertex) as within-subjects variables. Mauchly’s test indicated that the assumption of sphericity has not been violated (*p*s > 0.23 for accuracy and *p*s > 0.31 for RT).

The ANOVA on mean accuracy scores revealed a significant main effect of task condition, *F*(1, 17) = 19.06, *p* < 0.001, η_p_^2^ = 0.53, but not of TMS, *F*(2, 34) < 1, *p* = 0.42. Critically, the interaction task condition by TMS was significant, *F*(2, 34) = 7.53, *p* = 0.002, η_p_^2^ = 0.31. The significant two-way interaction was investigated by looking at the simple main effect of TMS within each task condition. TMS significantly affected detection of irregularities of new sequences, *F*(2, 34) = 4.69, *p* = 0.016, η_p_^2^ = 0.22. In particular, post-hoc comparisons showed that cerebellar TMS impaired participants’ accuracy compared to both early visual cortex TMS, *t*(17) = 2.63, *p* = 0.018 (with Bonferroni-Holm correction, *p* = 0.054) and vertex TMS, *t*(17) = 2.34, *p* = 0.032 (with Bonferroni-Holm correction, *p* = 0.064). The effect of TMS over the early visual cortex and vertex was comparable (*p* = 0.70). In turn, TMS did not affect detection of irregularities of familiar sequences, *F*(2, 34) = 1.38, *p* = 0.26.

The ANOVA on mean correct RT revealed a significant main effect of task condition, *F*(1, 17) = 22.95, *p* < 0.001, η_p_^2^ = 0.57, indicating that participants were faster (mean correct RT = 445 ms, *SD* = 47) in *familiar* compared to *new* sequences (497 ms, *SD* = 56). Neither the main effect of TMS, *F*(2, 34) = 1.87, *p* = 0.17, nor the interaction task condition by TMS, *F*(2, 34) < 1, *p* = 0.67, reached significance.

## Experiment 2

In Experiment 1, TMS selectively affected processing of new/unfamiliar sequences without affecting familiar sequences processing. The lack of cerebellar TMS effects on processing of familiar sequences supports the view that the cerebellum is differently involved in serial pattern detection depending on the familiarity of the sequence. Moreover, Experiment 1 ruled out the possibility that the detrimental effect of TMS depended on indirect stimulation of the visual cortex.

An important aspect to consider in interpreting results of Experiment 1 is that only the last item of the sequence could be violated. Hence, one may object that no effective serial processing was required, since in order to correctly perform the task one could just focus on the last item of the sequence. To investigate this possibility, we conducted Experiment 2 in which we presented a new group of participants with sequences composed by three geometrical shapes appearing consecutively as in the *new* sequences condition of Experiment 1. Hence, one of the three shapes composing the sequence was presented and participants had to indicate its order of appearance in the sequence (first, second or third). Importantly, to successfully perform the task, participants had to pay attention to all the elements of the sequence since they could all be potential targets. If the cerebellum plays a role in coding and maintain in memory the sequential order of the serial elements we should replicate the detrimental effect of cerebellar TMS over participants’ performance found in Experiment 1. In turn, if the cerebellum is involved in short-term memory of the single elements composing the sequence but not in their temporal relationship, cerebellar TMS should not effectively modulate participants’ performance.

### Methods

#### Participants

Eighteen Italian students (6 males, mean age = 23.1 years, *SD* = 2.3) participated in the experiment. None of them had participated in Experiment 1. Each participant filled in a questionnaire^27^ to evaluate their compatibility with TMS before undergoing the experiment. None of the participants reported neurological problems or history of seizures. None was taking medications that could interfere with neuronal excitability. Written informed consent was obtained from all participants before the experiment. The protocol was approved by the psychological ethical committee of the University of Pavia and participants were treated in accordance with the Declaration of Helsinki.

#### Material and Procedure

The experimental setting was the same as in Experiment 1. Stimuli consisted of the same visual shapes used in Experiment 1 but only the medium size (4*4 deg) was used. Each trial started with a central fixation cross (2500 ms) followed by the presentation of a sequence of three shapes. Each shape lasted for 500 ms and was separated by the consecutive one by a blank screen (200 ms). The last shape was followed by a blank screen (200 ms) and then by the presentation of the target shape (i.e., one of the elements of the sequence). Below each target shape an ordinal number (1^^^, 2^^^ or 3^^^) appeared. The number indicated in which order position the target element displayed above was presented in the just presented triplet (e.g., 2^^^ to indicate the second element of the sequence). In half of the trials, the number indicated the correct order position of the target element, whereas in the other half the number indicated an incorrect position. Importantly, the target element depicted with equal frequency all the order positions (first, second and last) of the sequence. In each trial, participants had to indicate by left/right key pressing with their right hand whether the number corresponded to the position in which the target element was presented in that sequence. Response keys assignment was counterbalanced among participants. The target shape remained on the screen until participants responded. Participants were instructed to respond as quickly as possible. Each block consisted of 108 trials and each participant performed the same block three times (once for each TMS site, see below). A practice session consisting of 16 trials was presented before the experiment.

#### TMS

TMS parameters and TMS sites were identical to Experiment 1. Triple-pulse 20 Hz TMS was delivered 150 ms before the presentation of the target element (so that the last TMS pulse was given 50 ms before the onset of the stimulus). Mean TMS intensity used was: 53.7% (SD = 4.0%). No participants reported phosphene perception during the experiment.

### Data Availability

All data collected in this experiment are available upon request.

### Results

**Figure 4:**
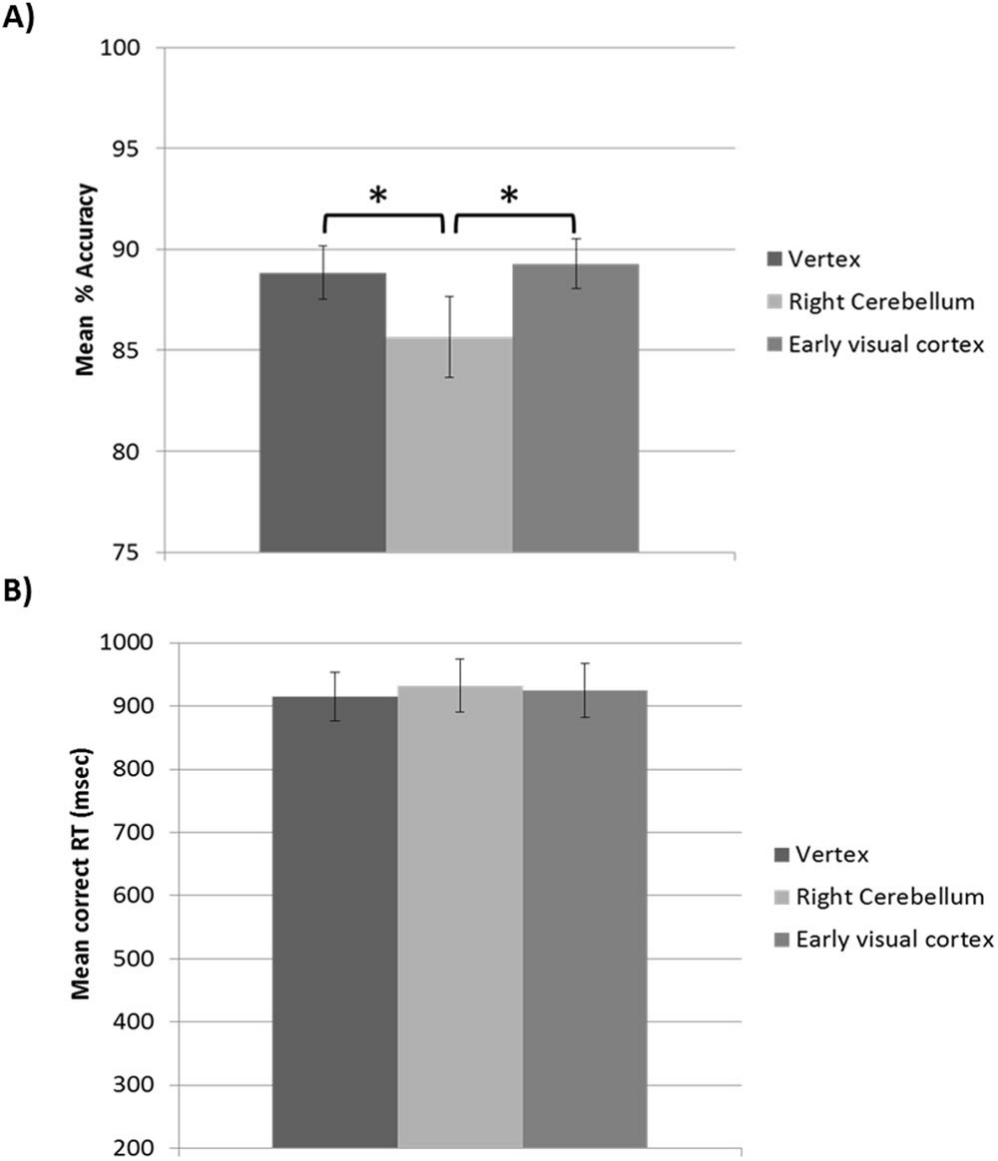
(**A**) Mean percentage accuracy scores and (**B**) mean correct RT as a function of TMS site (right cerebellum, early visual cortex and vertex) in Experiment 2. TMS over the cerebellum decreased participants’ accuracy rates compared to early visual cortex and vertex stimulation. TMS did not affect RT. Error bars represent ±SEM. Asterisks indicate significant differences between conditions (*p < 0.05).

Mean accuracy rates and mean RT for correct responses were computed for each participant in each TMS condition (Fig. 4). Accuracy and RT were analyzed via repeated-measures ANOVAs with TMS (cerebellum, early visual cortex and vertex) as within-subjects variable. Mauchly’s test indicated that the assumption of sphericity has not been violated (*p*s > 0.41 for accuracy and *p*s > 0.43 for RT).

The ANOVA on mean accuracy scores revealed a significant main effect of TMS, *F*(2, 34) = 4.12, *p* = 0.025, η_p_^2^ = 0.20, indicating that TMS impaired participants’ performance (see Fig. 4A). Post-hoc comparisons showed that accuracy rates were lower in the cerebellar TMS condition compared to both TMS over the early visual cortex, *t*(17) = 2.64, *p* = 0.017 (with Bonferroni-Holm correction, *p* = 0.051) and over the vertex, *t*(17) = 2.08, *p* = 0.053 (with Bonferroni-Holm correction, *p* = 0.11). The effect of TMS over early visual cortex and vertex was comparable, *t*(17) < 1, *p* = 0.72.

The ANOVA on mean correct RT revealed a non-significant main effect of TMS, *F*(2, 34) < 1, *p* = 0.18.

## Discussion

We found that TMS applied over the cerebellar hemisphere interfered with participants’ ability to memorize the order of appearance of a series of geometrical shapes (Experiment 1 and 2), without affecting processing of familiar alphanumeric sequences (Experiment 1). Importantly, this effect did not depend on indirect stimulation of early visual cortex.

Although our effects were overall of small size, they are in line with prior neuroimaging and neuropsychological evidence suggesting that the cerebellum is involved in processing perceptual sequences^10,14,15,17,45–49^. It is important to note that our experiments did not directly test sequence learning or violation that are typically assessed with deviant detection tasks (e.g., oddball paradigms, see Kotz *et al*., 2014^50^). Nonetheless, the storage in memory of the temporal association between incoming stimuli is critical to perceive events as linked in a sequence^23,50^. Moreover, predictive processes – in which the cerebellum seems to be involved - cooperate and actively build on mnemonic ones, helping to generate goal-directed and adapted behavior^23^.

In Experiment 1 only the last element of the sequence could vary between the *sample* and *test* sequences, and subjects could thus selectively focus on that to successfully perform the task (ignoring the preceding items). To control for this possibility, Experiment 2 measured participants’ ability to retain in memory the order of appearance of *all* the items of the series, forcing to process the whole sequence. Results of Experiment 2 suggest that the cerebellum is relevant in specifically coding the way stimuli are concatenated, in line with prior evidence^28,50–52^. In turn, cerebellar TMS did not affect recognition of familiar alphabetic or numerical progressive sequences. This supports prior neuroimaging findings showing preferential cerebellar response when participants had to decide whether the order of two probe stimuli (e.g., F, C) was consistent with that of a previous (just presented) sequence (e.g, D, C, I, F, J, A), but not when they had to decide whether the probes appeared in alphabetical or numerical progressive order^52^. Accordingly, in the motor domain, Doyon *et al*.^19^ found that the neural representation of a new sequence of movements becomes gradually less dependent on the cerebellum with learning and taps more onto striatal–cortical circuits^53^. In line with this, cerebellar patients are more impaired in procedural motor learning than in implementing sequences acquired via explicit instructions^17^. This is also paralleled by animal evidence showing that hemicerebellectomy in rats impairs the acquisition and execution of new spatial sequences but not the execution of sequences learnt before the lesion^54^.

In our experiments, we targeted cerebellar hemispheres and not medial structures in light of prior evidence indicating that perceptual and cognitive processing mainly occur in lateral vs. medial cerebellar regions^4,26^. Moreover, it is important to note that although TMS directly affects neurons in the targeted area, stimulation may also indirectly modulate neural responses in other proximal or distal regions (e.g., motor cortex or the prefrontal cortex^55,56^). Specifically, changes in the excitability of the cerebellar cortex may affect excitability of the Purkinje cells, hence modulation by deep cerebellar nuclei over cortical regions^41,57^. Although the lack of neuroimaging data in our study does not allow to disentangle whether our findings are due to direct effects of TMS on the cerebellar hemisphere or to more indirect effects, our data show that neurons located in the posterior lateral cerebellum contribute to the extended network underpinning visual sequence processing. Furthermore, our participants were overall faster and more accurate with familiar sequences, accuracy being overall higher than 95%. Ceiling performances are less likely to be modulated by TMS^58^ and cerebellar involvement in cognitive tasks has been found to depend on the complexity of the patterns to be processed^14,59,60^, response uncertainty^25^, and cognitive load^61^. In line with this, it remains to be investigated whether the cerebellum is involved also in retrieving familiar material when the task is sufficiently demanding.

In sum, by showing that the cerebellum plays a role in short-term memory for the order of incoming visual stimuli, our data contribute to a broader investigation on cerebellar involvement in extracting deterministic and probabilistic regularities in incoming sensory information (e.g., sequence detection) and in using this information to generate predictions about future events^15,62^.
